# Applications of the Novel Quantitative Pharmacophore Activity Relationship Method QPhAR in Virtual Screening and Lead-Optimisation

**DOI:** 10.3390/ph15091122

**Published:** 2022-09-08

**Authors:** Stefan Michael Kohlbacher, Matthias Schmid, Thomas Seidel, Thierry Langer

**Affiliations:** Division of Pharmaceutical Chemistry, Department of Pharmaceutical Sciences, University of Vienna, Josef-Holaubek-Platz 2, 1090 Vienna, Austria

**Keywords:** pharmacophore, pharmacophore modelling, quantitative pharmacophore, QSAR, machine learning, pharmacophore optimisation, NeuroDeRisk

## Abstract

Pharmacophores are an established concept for the modelling of ligand–receptor interactions based on the abstract representations of stereoelectronic molecular features. They became widely popular as filters for the fast virtual screening of large compound libraries. A lot of effort has been put into the development of sophisticated algorithms and strategies to increase the computational efficiency of the screening process. However, hardly any focus has been put on the development of automated procedures that optimise pharmacophores towards higher discriminatory power, which still has to be done manually by a human expert. In the age of machine learning, the researcher has become the decision-maker at the top level, outsourcing analysis tasks and recurrent work to advanced algorithms and automation workflows. Here, we propose an algorithm for the automated selection of features driving pharmacophore model quality using SAR information extracted from validated QPhAR models. By integrating the developed method into an end-to-end workflow, we present a fully automated method that is able to derive best-quality pharmacophores from a given input dataset. Finally, we show how the QPhAR-generated models can be used to guide the researcher with insights regarding (un-)favourable interactions for compounds of interest.

## 1. Introduction

Pharmacophore modelling was popularised at the turn of the millennium with increasing computational power and its general accessibility for researchers in the field of medicinal chemistry [1,2,3]. Since then, it has become an integral part of the methodological toolbox for computer-assisted drug discovery and design [4]. In the absence of a crystal structure, ligand-based pharmacophore modelling is often used in combination with the virtual screening of large compound databases in order to identify novel active compounds for a particular target of interest [5]. Even though many drug discovery success stories can be reported [6,7,8] where pharmacophore-based virtual screening was used as a key technology, the pharmacophore modelling process itself is often tedious, highly complex, error-prone, and relies heavily on the expert knowledge of the researcher. Various unknowns in pharmacophore modelling even often yield completely different results when applying different programs to the same dataset [9,10]. 

Before the 2000s, Chen et al. [11] proposed a system that analyses a dataset of a few thousand compounds and then generates suggestions for pharmacophore models based on the obtained knowledge. The presented method is a first step toward generating a system that analyses a set of data too complex for humans to fully grasp and present the obtained solutions to the researcher, who merely needs to decide on the best solution. We think machine learning has huge potential in computer-assisted drug discovery to achieve exactly that; analysing complex data to assist the researcher and offer guidance with the obtained solutions. 

Furthermore, Chen et al. pose two arguments contrary to popular heuristics applied in pharmacophore modelling. First, they state that weak or lesser active compounds contain important information for pharmacophore modelling. This argument contrasts the often practised method of selecting a highly active subset of compounds for pharmacophore modelling [5]. Nowadays, this is often considered by adding exclusion volumes to the pharmacophore. The second argument Chen et al. bring forward is that selecting an activity cutoff for active and inactive compounds is highly subjective and not clearly defined. Indeed, the cutoff may depend on factors such as the available dataset, and multiple experts might independently end up with various cutoffs for a certain dataset. Considering these arguments, the logical next step is the generation of pharmacophores from continuous data without the need for arbitrary choosing activity cutoff values. 

In addition to automated pharmacophore modelling, scoring and prioritisation of the obtained hits are not possible with the qualitative nature of pharmacophores. Consensus scoring [12] with multiple models is an often applied first step to solving this problem. Another solution is ranking the obtained hits by an external regression model. Considering that most consensus scoring methods are still qualitative in their nature and regression represents a different type of model, a combination of these two would be ideal for ranking the obtained hits. Eventually, this results in a method that prioritises hits with a previously validated pharmacophore model by assigning continuous activity values to the compounds. Combined with an automated approach to generate pharmacophore models from a given dataset containing only a few compounds, a researcher could quickly generate a prioritised list of hits for biological testing in the drug discovery campaign. 

In this paper, we present a novel method for automated pharmacophore modelling given a previously trained and validated QPhAR [13] model. We show that it outperforms the commonly applied heuristics for pharmacophore model refinement and can reliably generate a set of three-dimensional (3D) pharmacophores that show high discriminatory power in the virtual screening process. Combined with the training of a QPhAR model, we propose a fully automated workflow for generating a QPhAR model from a set of given compounds, deriving a classification-performance optimised pharmacophore (in the following referred to as ‘refined’ pharmacophore), using the pharmacophore for the virtual screening of molecule databases, and finally ranking the obtained hits by their predictions made with the QPhAR model. In addition, we highlight a method to visualise the expected changes in the activity of a compound when introducing certain pharmacophore features. The expected activity changes are displayed in a grid around the investigated compound, guiding the researcher with highlighted regions of favourable and unfavourable interactions. The proposed method and workflow aim at the analysis of, for human researchers, usually non-obvious information contained in ligand datasets and the presentation of this information in an easy-to-comprehend way. The expert user can then engage in decision-making based on the presented results of the performed analyses. 

## 2. Results and Discussion

We conducted a case study on the hERG K+ channel using the dataset from Garg et al. [14] and the correspondingly trained QPhAR model. First, we will discuss the process of generating a refined pharmacophore and its comparison against established baseline methods. Second, on the basis of the hERG example, we describe how the information provided by a QPhAR model can be utilized in a fully automated end-to-end pharmacophore modelling workflow. Finally, we will close the discussion with a few examples of how QPhAR can be used to guide a medicinal chemist to further insights after a set of compounds has been selected from an obtained virtual screening hit list. 

### 2.1. Generation of a Refined Pharmacophore for Virtual Screening

**Figure 1:**
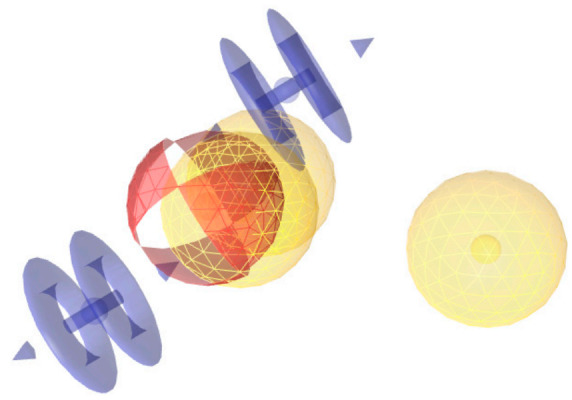
Refined pharmacophore of “Garg et al.” [14] dataset.

For each dataset investigated, we have applied the devised algorithm to extract refined pharmacophore features from the QPhAR model. The pharmacophore can be generated directly from the model without the requirement of additional data. Therefore, all molecules contained in the datasets can be used to evaluate the generated pharmacophore. Nevertheless, it makes sense to keep the training-test split of each dataset for a final validation of the selected models on the test set. Following this strategy, the generated pharmacophores were evaluated on the training set, ranked by their *F_β_*-score and *F_Specificity_*-score, and the top five models validated on the provided test set. Figure 1 shows the refined pharmacophore model generated for the dataset obtained from Garg et al. [14].

In contrast to the generation of refined pharmacophores, the generation of shared pharmacophores, the baseline method, requires an input dataset. Shared pharmacophores were chosen as the baseline for two reasons. First, shared feature pharmacophore generation is often employed as the “first-in-line” method when it comes to ligand-based pharmacophore modelling. Second, pharmacophores of highly active compounds are assumed to contain many features of relevance for high compound binding affinity. The baseline models were generated from the *n* most active compounds in the training set, with *n* serving as a hyperparameter. These pharmacophores were validated on the training and test set in the same manner. The results and a comparison against the performance of the refined pharmacophores can be found in Table 1. 

The baseline and QphAR-based refined pharmacophores were scored and compared using the *F_Composite_*-score. The typical metrics used in machine learning, such as accuracy, precision, sensitivity, etc., are not accurately depicting the situation in virtual screening. Scoring pharmacophore models with these metrics would lead to results which might not be considered optimal in this context. Often the objective is to get as many true positives as possible while reducing the number of false positives. The number of false negatives can often be ignored with the reasoning that a missed hit does not consume any resources, whereas false positives will. Accuracy and others are not considering these objectives and put the same emphasis on both numbers. It should be noted that the ROC-AUC score is often used in virtual screening experiments and does reflect the objective much better than accuracy and others. However, due to the ROC-AUC score’s non-linearity, we think it often gives the perception of the results being better than they are. Therefore, we used the *F_β_*-score, *F_Specificity_*-score, and *F_Composite_*-score to score the obtained pharmacophore models. 

As can be seen in Table 1, the QPhAR-based refined pharmacophores score better than the baseline pharmacophores on the *F_Composite_*-score, although a dependency on the quality of the QPhAR models can be observed. The lower the performance of the QPhAR model, the less reliable it is in generating a refined pharmacophore. This, however, is not surprising since the quality of the workflow we describe here depends heavily on the trained QPhAR model. Therefore, we advise the user to emphasise training and validating the QPhAR models to increase the model’s performance and narrow the confidence interval.

### 2.2. End-to-End Pharmacophore Modelling

**Figure 2:**
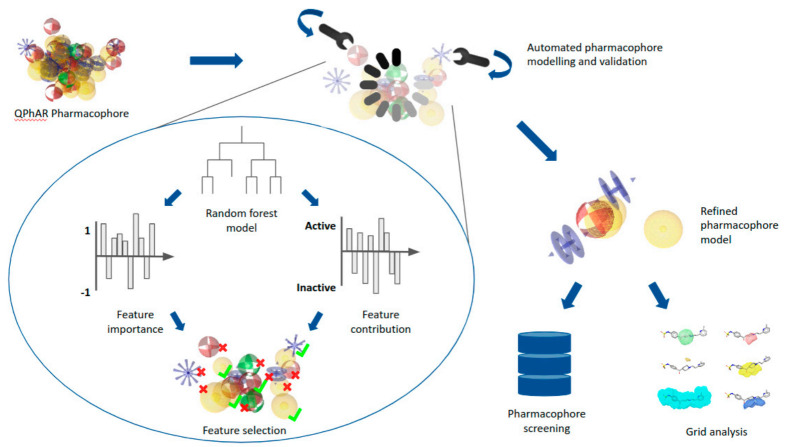
Schematic depiction of end-to-end pharmacophore modelling workflow.

Applying the aforementioned algorithm to generate refined pharmacophores from QPhAR models, we developed a workflow (Figure 2) for fully automated pharmacophore modelling, virtual screening and ranking of the obtained hits. The workflow is completely ligand-based; therefore, only a small set of compounds of ~15–50 ligands with known activity values is required. We will assume IC50 or Ki values here, although theoretically any physicochemical property can be used. 

The first step is to prepare and clean a dataset for the target of interest. Here, we use the dataset published by Garg et al. [14] on the widely known hERG K+ channel. The dataset is split into a training and test subset (we adopt the splitting ratio provided in their publication), and a QPhAR model is generated using the training set molecules. The QPhAR model is validated on the before separated test set using cross-validation, leave-one-out analysis, y-scrambling and a paired t-test (results have been published previously [19]). Afterwards, the refined pharmacophore model is generated using the procedure outlined in the methods section. The refined pharmacophore is then validated on the separated test set before being used to screen a database of virtual molecules. We use a filtered version of the Molport database containing ~1.25 million molecules. Since pharmacophore-based virtual screening is only a qualitative method, it is not possible to directly prioritise some compounds from the hit list over others on the basis of particular physicochemical properties of interest (e.g., their IC50 value). Therefore, the next and final step is to score and rank the obtained hit list (14871 molecules, ~1% hit-rate) with the previously trained QPhAR model. The obtained ranked hit list is provided as an SD-file in ascending order of relevance (highest activity value first). The data can be found along with the remaining data in the author’s GitHub repository (https://github.com/StefanKohlbacher/qphar-applications). 

Even though the entire workflow can be automated from start to end, we recommend including sanity checks at certain key events, such as validating the trained QPhAR model performance and the completed generation of the derived refined pharmacophore. For both steps, we suggest to define key metrics and corresponding values that should be fulfilled before the workflow proceeds.

### 2.3. Three-Dimensional Pharmacophore Activity Profiling

Finally, the hit-list obtained from the end-to-end pharmacophore modelling workflow will serve as a starting point for medicinal chemists to further optimise compounds in the hit-to-lead phase of the drug discovery pipeline. Once a few promising compounds have been identified, the main question to be answered is: “What modifications should be introduced to the molecule to improve its affinity, solubility, bioavailability, etc.?” Some of these properties will depend more on the target that is being investigated than others. For example, affinity should always be considered in the context of the structure of the target receptor. We will conclude the end-to-end pharmacophore modelling workflow with a ligand-based approach that guides the medicinal chemist in this process and provides him with insights and ideas for reasonable structure modifications steps. 

As explained in detail in the methods section, the QPhAR model may be used to generate 3D-activity grids around a molecule or pharmacophore. Grids can be generated for each feature type present in the QPhAR model and will be split into positive and negative contributions. The positive grids can be interpreted as points in space, where a pharmacophore feature of this type would be beneficial for a higher activity of the given molecule towards the target receptor. Such kind of information is invaluable for any medicinal chemist working on the structural optimisation of lead compounds. It provides the location as well as the type of interaction that potentially improves the sought-after property of an investigated molecule. Negative grid regions, on the other hand, can be interpreted as portions of space where features of a particular type are unfavourable. Any feature of the analysed type in this region is expected to reduce the molecule’s activity towards the target and should be avoided, if possible. Unless the model is generated for an anti-target, such as hERG. In such cases, the negative grids might provide the medicinal chemist with ideas on optimising a molecule’s structure in a way that helps to avoid binding to the anti-target.

To elaborate on that, we analysed the activity grids of selected known hERG blockers to explore the potential of this method. The blockers were obtained from Perry et al. [20], whereas two of these are discussed in further detail here. Molecules, pharmacophores, as well as generated activity grids, are provided in the data in the author’s GitHub repository (https://github.com/StefanKohlbacher/qphar-applications). 

**Figure 3:**
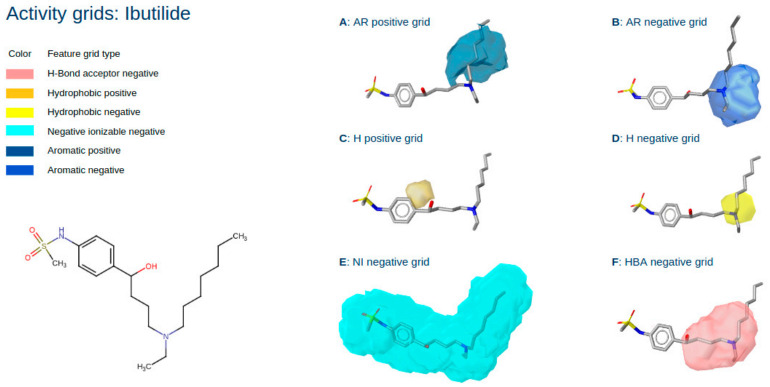
Structure of the known hERG blocker Ibutilide and its respective activity grids: aromatic positive grid (A); aromatic negative grid (B); hydrophobic positive grid (C); hydrophobic negative grid (D); negative ionizable grid (E); hydrogen bond acceptor negative grid (F).

Figure 3 shows the generated activity grids for Ibutilide, a known hERG blocker. For each of the six pharmacophore feature types (Aromatic—AR, Hydrophobic—H, H-Bond acceptor—HBA, H-Bond donor—HBD, Positive ionizable—PI, Negative ionizable—NI), a positive and a negative grid was generated. Only the grids relevant to Ibutilide are shown.

The hERG channel has a well-studied ligand SAR with known distinct binding features that are relevant for high activity. These are two aromatic features, although one is sufficient for strong binders, and a basic nitrogen, forming a y-shaped binding motive [21]. As a rule of thumb, the more hydrophobic a compound is and the lower its pKa, the more likely it will bind to the hERG channel. Figure 3A,B show the grids for aromatic features. Both grids provide information on how the activity of Ibutilide towards hERG is expected to change when introducing a feature (functional group) within the outlined locations. Improvements in activity can be expected when an aromatic feature is introduced at the aliphatic chain neighbouring the basic nitrogen. The positive field shows a clear distinction to locations near the nitrogen, where an aromatic feature would be unfavourable, as seen in the negative aromatic field, which colocates the basic nitrogen. Introducing an aromatic feature in the aliphatic chain would nicely match the known SAR of the y-shaped binding motive. Furthermore, Figure 3C,D show the activity fields for additional hydrophobic features introduced to Ibutilide. Here, introducing a hydrophobic feature near the phenyl ring (Figure 3C) would yield positive results, as expected due to the fact that hydrophobicity generally increases the affinity to hERG. On the other hand, the negative field in Figure 3D indicates that introducing a hydrophobic feature near or instead of the basic nitrogen would lead to a decrease in expected activity. Again, this agrees with the known SAR, highlighting the central nitrogen’s basicity as a crucial binding motive. Similarly, introducing a negative ionisable feature, such as a carboxylic acid, to any location in the molecule increases the pKa, which is known to be unfavourable. This fact can be observed in Figure 3E. Finally, there is the negative field of H-bond acceptor features shown in Figure 3F, which indicates negative expected changes when introduced at or near the basic nitrogen atom. The conclusions obtained from this field are not as clear as those from the other fields. On the one hand, introducing H-bond features, replacing some of the hydrophobic interactions, would decrease the logP, which is roughly equivalent to an increase in pKa and, therefore, unfavourable for binding. On the other hand, H-bond acceptors would be able to interact with external hydrogens in a similar fashion as the positive ionisable group from the basic nitrogen. The strength of this interaction and, therefore, the activity depends heavily on the functional group introduced. Therefore, it is not immediately clear that introducing an HBA feature would result in a negative expected change of activity. 

**Figure 4:**
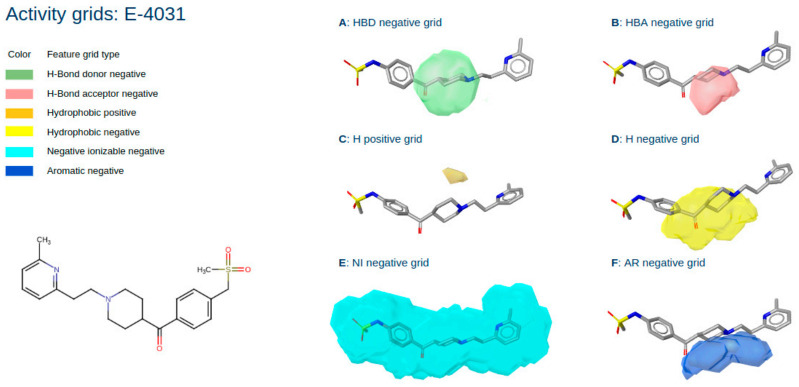
Structure of the known hERG blocker E-4031 and its respective activity grids: hydrogen bond donor negative grid (A); hydrogen bond acceptor negative grid (B); hydrophobic positive grid (C); hydrophobic negative grid (D); negative ionizable grid (E); aromatic negative grid (F).

A similar analysis can be made for the molecule E-4031 shown in Figure 4, which is also a known hERG blocker. For subparts Figure 3B–F, the conclusions drawn are the same as those discussed above for Ibutilide, which shows a clear agreement of its 3D activity profile with the known SAR of hERG as reported in the literature [21]. Additionally, Figure 2A shows an activity field for expected negative interactions with H-bond donors colocated with the basic nitrogen atom of E-4031. It follows that replacing the basic nitrogen would be detrimental to activity since the opposing interaction partner is expected to donate a hydrogen atom to the nitrogen, forming an ionic interaction. Opposing such an interaction with another H-bond donor on the side of E-4031 would lead to a loss of this ionic interaction, clearly unfavourable for high-affinity binding to hERG. 

Overall, Figure 3 and Figure 4 nicely show that an analysis of selected ligands with the QPhAR model derived grids can provide valuable insights for a medicinal chemist and provide him with ideas and even clear directions for optimising the hit or lead molecules. An additional case study based on the dataset from Ece et al. [15,22,23] can be found in the supplementary material. 

## 3. Materials and Methods

The algorithm and workflows described in the following were implemented, unless stated otherwise, in Python 3 using functionality provided by the Chemical Data Processing Toolkit [24].

### 3.1. Datasets and Training of QPhAR Models

The selection of datasets for quantitative studies is not straightforward and often underestimated. Here, we chose datasets that already have been used in previous validation studies [19] of the QPhAR algorithm. Nevertheless, the datasets were required to fulfill the following criteria:

- A separate training and test set has been defined previously.
- The training set contains between 15 and 30 molecules.
- Activity values for each compound in the dataset were measured in Ki or IC50 values.
- To avoid modelling experimental noise, the associated activity values range by at least three orders of magnitude.

Finally, the activity values have to be somewhat homogeneously distributed over the dataset and not clustered. This requirement has been validated visually. 

After filtering, five datasets remained, which were used to evaluate the developed workflows and methods. The datasets are provided for download on the author’s Github repository (https://github.com/StefanKohlbacher/qphar-applications). Three-dimensional conformations were calculated for each dataset using LigandScout’s iConfGen [25]. Default settings were used with a maximum of 25 output conformations for each molecule. Training and test data were split as described in the publications associated with the datasets [14,15,16,17,18,22,23]. Each compound in each dataset was categorised into active and inactive. As a default, the compounds were ranked by their activity, with the compounds in the top 20th percentile being labelled as active, and the remaining compounds as inactive.

### 3.2. Screening Baselines

Shared-pharmacophore models were generated and used as baselines in this study. They were generated from a subset of active compounds for each dataset based on typical assumptions made in pharmacophore modelling [5,26]. Whether a compound is considered active or inactive strongly depends on the context of the investigated target and often requires in-depth knowledge about its peculiarities. Usually, values in the range of 1 µM are considered a reasonable threshold for the separation into actives and inactives. The analysed datasets contained compounds ranging from a few nM to a few hundred µM. Therefore, and due to the relatively homogeneous distribution of activity values in the datasets, the cutoff for active compounds was set at the 20th percentile of the dataset. Any compound with activity values below this threshold was considered active, all other compounds inactive. This subset was subsequently used to generate a shared-pharmacophore with LigandScout’s [25] command-line tool Espresso.

### 3.3. Hyperparameter Optimisation

Hyper-parameters were optimised both for the generation of the refined pharmacophore and the shared-pharmacophore baseline (number of most active compounds to use for the generation of the shared-pharmacophore). The following parameters were optimised for the refined pharmacophore:

- Weight features by importance: True, False.
- Set exclusion volumes: True, False.
- Calculate feature contribution from ML (alternatively from QPhAR model): True, False.
- Number of resulting features: [4, 8].

### 3.4. Refined Pharmacophore Generation Algorithms

In the following, the algorithm to generate a refined pharmacophore from a trained QPhAR model will be explained in detail. The algorithm is based on the assumption that the QPhAR model was trained using a random forest (RF) regressor. Random forest was chosen since it has been shown to be the most promising method to train a QPhAR model [19]. However, similar conclusions can be derived from other machine learning models, such as linear regression models. 

The generation of a refined pharmacophore in the QPhAR context consists of four main steps:

- Determination of feature importance.
- Determination of feature contribution.
- Processing negatively contributing features.
- Selection of features for refined pharmacophore.

### 3.5. Determination of Feature Importance

Feature importance is derived from the underlying machine learning model of QPhAR via extraction from the random forest model generated by scikit-learn’s [27] RF implementation. The feature importance is calculated during the training of the machine learning model and gives insight into the amount of information provided by this feature. The higher the feature’s importance, the more information it contains, and the more relevant it is for activity prediction. An analogous concept would be the set of coefficients in a linear regression model. 

### 3.6. Determination of Feature Contribution

In contrast to the feature importance, which is easily obtained, the information on whether a feature contributes positively or negatively to predicted values is not immediately accessible in RF-based models. Within the context of a trained QPhAR model, this information can be obtained directly from the QPhAR pharmacophore without additional information from the machine learning model.

- Feature contribution information derived from the QPhAR pharmacophore model: As explained in the QPhAR publication [13], the QPhAR algorithm associates each newly generated pharmacophore feature with a list of activities. These activities will not only be used to determine the relevance of the feature—whether it is actual information or just adds noise to the model—but also to determine the contribution of a pharmacophore feature to the models’ predictions. The mean activity based on the list of associated features is calculated for each feature, resulting in one feature-activity for each pharmacophore feature. Finally, the feature-activities are compared against each other and scaled by their variance. Features with a positive sign of its scaled activity are considered to contribute positively to the prediction of the QPhAR model. Features with a negative sign contribute negatively to the prediction.
- Feature contribution information derived from the RF model: To extract feature contributions from an RF model in a deterministic way, two assumptions are made. First, the data provided to the machine learning model in the QPhAR algorithm represent the pairwise distances between features of the QPhAR model and the pharmacophore to predict. Second, applying the splitting criterion of each node in a tree of the random forest model will yield the left-child node for input values below or equal to the splitting threshold and the right-child node for input values above the splitting threshold. Both these assumptions are ensured by the implementation of the QPhAR algorithm as well as scikit-learn’s RF implementation.
- Following this logic, a simple algorithm can be devised to determine whether a feature contributes positively or negatively to the prediction of a sample. For each node in each tree, the node’s value is obtained and compared against its neighbouring node. Suppose the left child node has the higher predicted activity. In that case, we can assume that this feature contributes positively to activity since the left child node represents a smaller distance of pairwise pharmacophore features. At the same time, the right child node yields the lower activity prediction, which is associated with a larger distance of pharmacophore feature pairs. On the other hand, if the left child node yields the lower predicted activity, which is associated with a smaller feature pair distance, then the feature can be considered to contribute negatively to activity.
- During this process, the feature-id of each node is obtained, which corresponds to the pharmacophore feature it represents. The value of the feature with the corresponding feature-id is aggregated as the mean value of all nodes that either obtain their value from this feature-id or have a child node that obtains the prediction processing this feature-id. Once all trees and nodes are processed, a value representing the activity is obtained for each pharmacophore feature. These values are scaled as above by their variance. Once again, features with a positive sign are considered to contribute positively to the activity, whereas features with a negative sign are considered to contribute negatively to the activity.

### 3.7. Processing Negatively Contributing Features

Based on the analysis of feature contribution in the previous step, a post-processing step for negatively contributing features is carried out. The algorithm includes the option to either ignore these features entirely, in which case they are removed from the refined output pharmacophore, or convert them to exclusion volume spheres. 

### 3.8. Selection of Features for the Refined Output Pharmacophore

Finally, the output pharmacophore is created from this list of features with their associated activity values. The features are sorted by their activity contribution values in descending order, resulting in the feature with the most positive contribution in the first place. If feature importance have been obtained from a random forest model in the next-to-last step, the features can optionally be weighted by their feature importance. The first *x* features are then added to the output pharmacophore, whereas *x* is a value specified by the user beforehand and the value of the feature is not negative. *x* is recommended to be a value within the interval [4, 8]. If exclusion volume spheres have been generated in the previous step, these are also added to the refined output pharmacophore based on the sorted list of features. 

### 3.9. 3D Activity Profiling

The activity profile of a sample, pharmacophore or molecule, in 3D space, can be generated with the help of a previously trained QPhAR model. The model should be validated sufficiently before its use and have a narrow confidence interval for high confidence in the model’s predictions. The sample of interest is then aligned to the QPhAR model, and the baseline prediction is obtained. A grid is generated with a predetermined interval and some margin extending the sample’s size. For each pharmacophore feature type, a probe is placed and moved along the grid. At each point, the current pharmacophore is predicted by the QPhAR model, and the prediction is associated with the location in the grid. Once all grid points are processed, the differences between the predicted grid point values and the previously obtained baseline prediction are calculated. Optionally, the obtained grid of differences can be normalised for better analysis. 

The grids were saved in the *.kont format and then loaded into LigandScout alongside the molecules and pharmacophores for analysis. The terms ’activity grids’ and ‘activity fields’ will be used interchangeably in the remainder of this section. 

### 3.10. Metrics

The F1-score, or F-score, is a well-known and often applied metric in machine learning [28] and is defined as the harmonic mean of precision and sensitivity. However, due to the nature of virtual screening, the following scores, derived from the F1-score, will be more suitable for characterising the results of this study. 

#### 3.10.1. *F_β_*-Score

The *F_β_*-score [29] is directly derived from the F1-score and weights precision and sensitivity by the factor *β*. It is calculated by

(1) $$F_{\beta }=\frac{\left(1+\beta^{2}\right)*precision*recall}{\beta^{2}*precision+recall},$$

The *β*-value was set to 0.5 for all evaluations in this study.

#### 3.10.2. *F_Specificity_*-Score

Analogous to the *F_β_*-score, we define the *F_Specificity_*-score to focus more on the ratio between false positive and false negative hits during virtual screening.

(2) $$F_{Specificity}=\frac{precision*specificity}{precision+specificity},$$

#### 3.10.3. *F_Composite_*-Score

We define the *F_Composite_*-score, which is calculated as the mean of the *F_β_*-score and *F_Specificity_*-score, as a metric to model the objective of virtual screening.

(3) $$F_{Composite}=\left(F_{\beta }+F_{Specificty}\right)/2,$$

## 4. Conclusions

Nowadays, pharmacophore-based methods can be considered indispensable and are an integral part of nearly every modern computer-aided drug design project. A combination of pharmacophore modelling and pharmacophore-based virtual screening is often applied as one of the first filtering techniques to obtain a list of promising compound candidates for biological testing in the hit-finding phase. Despite its popularity, pharmacophore modelling is still a task that heavily relies on the expert knowledge of the researcher. In this study, we presented a method for the generation of pharmacophore models with high discriminatory power from a QPhAR model in a deterministic manner following clear generation guidelines. We showed that the pharmacophores derived by our algorithm are superior to a baseline of ligand-based pharmacophore models generated under the assumption that only active molecules are required to produce good query pharmacophores for virtual screening. Furthermore, we incorporated the presented method into a workflow for end-to-end pharmacophore modelling. This workflow facilitates a fully automated process to train a QPhAR model, generate a query pharmacophore from this QPhAR model, screen a database, and finally rank the obtained hits by relevance using the initial QPhAR model. In a case study using known hERG K+ channel blockers, we have shown that the generated activity fields agree well with the known SAR and can, therefore, provide meaningful insights for medicinal chemists in the hit or lead-optimisation phase. 

## Figures and Tables

**Table 1:** Test performance of the shared pharmacophore baseline models and refined pharmacophores obtained from the corresponding QPhAR models.

Data Source	*F_Composite_*-Score		QphAR Model Performance	
	Baseline	QphAR	R^2^	RMSE
Ece et al. [15]	0.38	0.58	0.88	0.41
Garg et al. [14]	0.00	0.40	0.67	0.56
Ma et al. [16]	0.57	0.73	0.58	0.44
Wang et al. [17]	0.69	0.58	0.56	0.46
Krovat et al. [18]	0.94	0.56	0.50	0.70

## Data Availability

Data are contained within the article and supplementary material.

## Supplementary Materials

The following supporting information can be downloaded at: https://www.mdpi.com/article/10.3390/ph15091122/s1, Figure S1: Structure of the known CDK2 inhibitor Flavopiridol and its respective activity grids; Figure S2: Structure of the known CDK2 inhibitor Roscovitine and its respective activity grids.
