# Supplementary material for: Applications of the Novel Quantitative Pharmacophore Activity Relationship Method QPhAR in Virtual Screening and Lead-Optimisation
Source: Pharmaceuticals (Basel). 2022 Sep 8;15(9):1122. doi: 10.3390/ph15091122 (PMC9504690; doi:10.3390/ph15091122)
Supplement: Supplementary file 1 [file pharmaceuticals-15-01122-s001.zip › pharmaceuticals-1863617-supplementary.pdf]

Applications of the novel quantitative pharmacophore activity relationship method QPhAR in virtual screening and lead-optimization

Stefan M. Kohlbacher, Matthias Schmid, Thomas Seide and Thierry Langer

Supplementary Material

An additional case study for activity profiling has been carried out on the target CDK2 with the model obtained from Ece et al. [15]. The analysed molecules were obtained from Zhang et al. [16]. A few characteristic binding motives and relationships have been identified in previous SAR studies [17]. A few of these insights are:

- An increase in the hydrophobic surface area of the ligand generally leads to reduced biological activity
- H-Bonds to the protein backbone are crucial for binding

If available, further insights on the ligand-protein interaction were obtained from inspecting the co-crystallized ligand in the binding pocket. The activity grids, as well as aligned molecules and pharmacophores for all molecules, can be found on the author's GitHub repository.

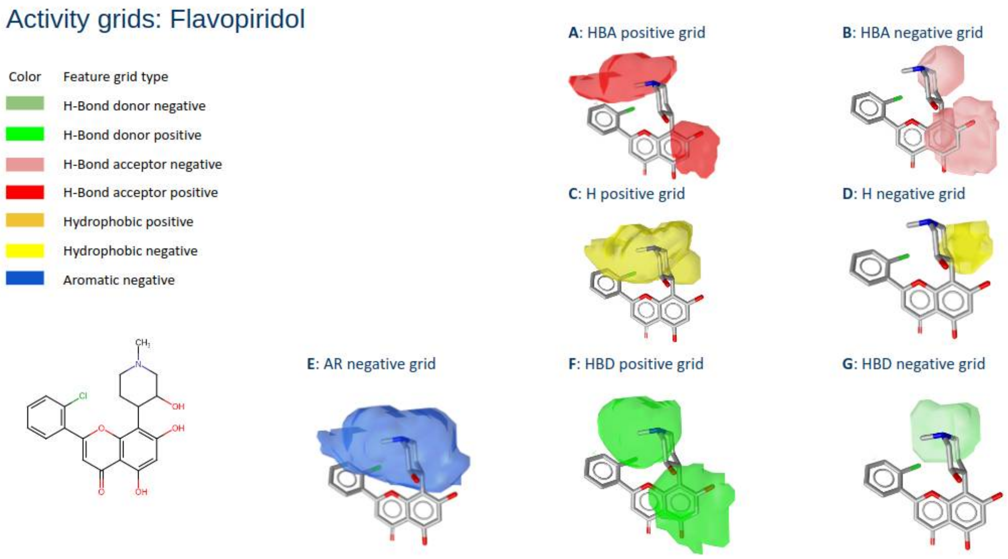

Figure S1. Activity grids for the known CDK2 inhibitor Flavopiridol.

Figure S1 shows the obtained activity grids for Flavopiridol, a strong CDK2 inhibitor, with a reported K_d of 23 nM in the PDB entry of 6GUB. The crystal structure (PDB-Code: 6GUB) was used to gain further insights into favourable interactions and compare these insights against the information provided by the activity grids.

The grids of Figure S1 **A** and **B** show the expected positive and negative regions, respectively. Considering the overall SAR knowledge as well as the supposed position of the molecule in the binding site (Note: The pose of the molecule in the binding site is similar but different from the aligned pose of the molecule to the QPhAR model. These incongruencies between the two conformations and their independent positioning, docking and pharmacophore alignment,

unfortunately, do not allow for a direct comparison of the grids within the binding site.), the positive HBA grids agree very well with the known SAR motives. An interesting case that should be highlighted is the almost overlapping positive and negative field on the right-hand side of the molecule. However, when inspecting the grids in 3D, it becomes clear that the negative fields co-locate with the molecule's atoms, whereas the positive fields are further distant from the molecule, indicating the importance of spatial distance with H-bonding interactions. The hydrophobic fields, **C** and **D**, lead to similar conclusions. The negative field, which in the image is located in front of the molecule, agrees very well with known SAR and the protein binding site. The positive field is slightly ambiguous. On the one hand, it is located on the backside of the molecule near a valine residue of the protein, which agrees with the suggestion of the activity field. However, the size of the positive field is questionable. The negative AR field agrees well with known SAR. Finally, the positive and negative grids for HBDs shown in **F** and **G** agree well with the known SAR as well as the other overlapping fields. For example, on the backside of the molecule, the negative HBD fields and the positive H fields are co-locating the same space. Again, given the molecules supposed orientation in the binding site and the vicinal valine residue, H-Bond donor interactions would be unfavourable in this position. On the other hand, the positive HBD fields indicate favourable expected interactions at the front of the molecule.

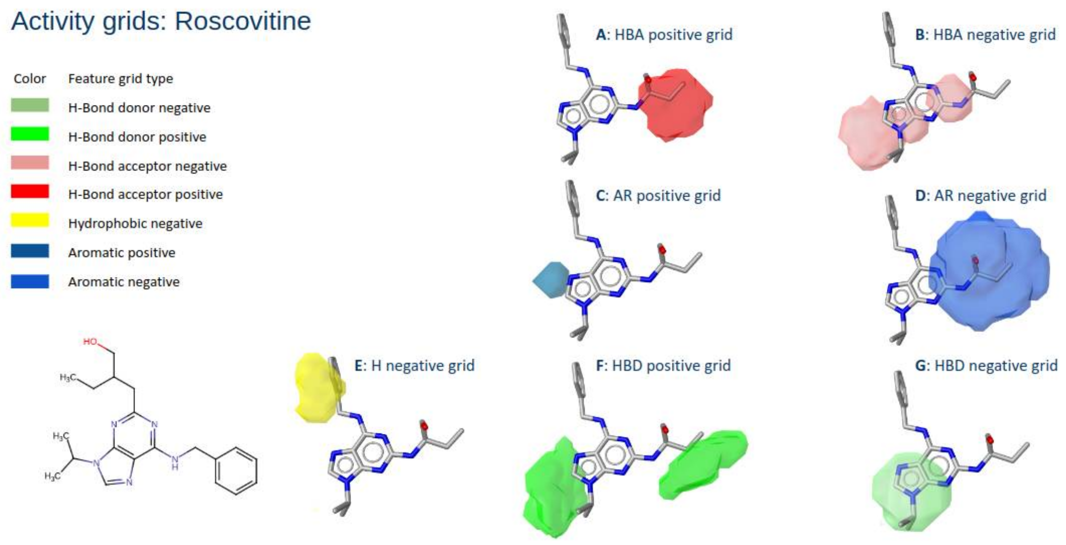

Figure S2: Activity grids for the known CDK2 inhibitor Roscovitine.

Figure S2 shows a similar analysis for the CDK2 inhibitor Roscovitine. Overall, similar conclusions, as were drawn for Flavopiridol, can be obtained for the activity grid analysis of Roscovitine. An interesting fact to highlight is the positive HBD grid. Inspection of the co-crystallized ligand in the binding site (PDB-Code: 3DOG) indicates a missing HBD interaction deep into the pocket. A typical binding motive for kinase inhibitors is the alternating HBD-HBA-HBD interaction of the ligand with the protein backbone. Roscovitine, however, is missing an HBD interaction inside the pocket. Analysing the positive HBD fields leads to the same conclusion, an additional HBD interaction is expected to be favourable for the molecule's activity. The conclusion is further supported by the negative HBA fields, which indicate unfavourable interactions when introducing an HBA feature in this region of the molecule.
